# *Salmonella enterica* serotype Typhimurium DT104 Isolated from Humans, United States, 1985, 1990, and 1996

**DOI:** 10.3201/eid0804.010202

**Published:** 2002-04

**Authors:** Efrain M. Ribot, Rachel K. Wierzba, Frederick J. Angulo, Timothy J. Barrett

**Affiliations:** Centers for Disease Control and Prevention, Atlanta, Georgia, USA

**Keywords:** multidrug resistance, class-1 integron, pulsed-field gel electrophoresis, R-type, ACSSuT, polymerase chain reaction, DNA sequencing

## Abstract

First isolated from an ill person in 1985, multidrug-resistant *Salmonella enterica* serotype Typhimurium DT104 emerged in the mid-1990s as a strain of *Salmonella* frequently isolated from humans in the United States. We compared the integron content, plasmid profile, and *Xba*I pulsed-field gel electrophoresis (PFGE) patterns of multidrug-resistant *S.* Typhimurium DT104 (MR-DT104) isolated from humans in the United States in 1985, 1990, and 1996. All isolates contained a 60-mDa plasmid and had indistinguishable PFGE and integron profiles, supporting the idea of a clonal relationship between recent and historical isolates. The data suggest that the widespread emergence of MR-DT104 in humans and animals in the 1990s may have been due to the dissemination of a strain already present in the United States rather than the introduction of a new strain.

Each year, bacteria of the genus *Salmonella* infect an estimated 1.4 million persons; these infections result in several hundred deaths in the United States annually (*1*). One of the most common strains isolated from humans is multidrug-resistant *Salmonella enterica* serotype Typhimurium definitive type 104 (DT104). This strain was first isolated from humans in 1984 in the United Kingdom, where it emerged as a major cause of human illness in the late 1980s (*2*) before its emergence in the United States and elsewhere in the mid-1990s (*3**,**4*). A national sample, in which all state and territorial public health laboratories were asked to forward every 10th *Salmonella* isolate to the Centers for Disease Control and Prevention (CDC) for antimicrobial susceptibility testing, showed that 275 (28%) of 975 *S.* Typhimurium isolates from humans in the United States in 1995 were resistant to ampicillin, chloramphenicol, streptomycin, sulfonamides, and tetracycline (R-type ACSSuT), the resistance pattern commonly associated with multidrug-resistant DT104 isolates (MR-DT104). In contrast, only 8 (7%) of 108 *S.* Typhimurium isolates from humans in sentinel counties in 1990 and 7 (5%) of 135 in 1985 were R-type ACSSuT. An isolate collected in 1985 probably represents the earliest isolate of MR-DT104 in the United States. After emerging in the mid-1990s, MR-DT104 has remained prevalent in the United States; in 1999, 114 (31%) of 362 human *S.* Typhimurium isolates received by the National Antimicrobial Resistance Monitoring System (NARMS) for Enteric Bacteria were R-type ACSSuT (NARMS 1999 Annual Report; http://www.cdc.gov/ncidod/dbmd/narms). Since Typhimurium was the most common serotype of *Salmonella* in the United States in 1999, causing 25% of the culture-confirmed infections (CDC Salmonella Surveillance, 1999 Annual Summary; http://www.cdc.gov/ncidod/dbmd/phlisdata/salmtab/), MR-DT104 caused an estimated 7% of *Salmonella* infections**.**

Food-producing animals are reservoirs for nontyphoidal *Salmonella*, and most human *Salmonella* infections in the United States are a consequence of eating food, particularly foods of animal origin, contaminated with *Salmonella* (*5*). In 1998, 163 (29%) of 557 *S.* Typhimurium isolates from animals received in NARMS were R-type ACSSuT; most of these isolates were collected from cattle and pigs (NARMS 1998 Veterinary Isolates Report; http://www.fda.gov/cvm/fda/mappgs/narms/1998_data/narms_toc.htm). Limited data are available, however, on the prevalence of MR-DT104 in food-producing animals before 1996. A recent report indicates that MR-DT104 became prevalent in cattle in the Pacific Northwest in the mid-1990s (*6*); 55 (45%) of 123 cattle *S.* Typhimurium isolates from 1995 to 1997 were R-type ACSSuT, compared with 18 (20%) 90 in 1991 to 1994 and 2 (1%) of 163 from 1985 to 1990. Isolates of MR-DT104 from animals and humans were indistinguishable by molecular subtyping techniques (*7*).

The genetic determinants responsible for the R-type ACSSuT in MR-DT104 are located in the chromosome (*2*,*8*). Molecular characterization of *S.* Typhimurium DT104 R-type ACSSuT isolates from Europe and the United States showed that resistance to ampicillin, streptomycin, and sulfonamides is associated with the presence of two class-1 integrons (*7*–*10*). First described by Stokes and Hall in 1989 (*11*), integrons are a group of apparently mobile elements that can contain one or more antimicrobial resistance genes. Integrons represent an important and efficient mechanism by which many bacteria, including *S.* Typhimurium DT104, can acquire resistance to antimicrobial agents. These genetic elements have been found in a wide variety of organisms and are thought to be largely responsible for the dramatic increase in multidrug-resistant bacteria (*11*–*15*). Integrons carrying antimicrobial resistance genes have been found in plasmids and transposons (and transposon-like elements) and in the chromosomal DNA of some bacteria. The fact that integrons are widely spread among gram-negative bacteria suggests that these genetic elements have evolved into a highly adaptable and very efficient mechanism by which cells can acquire and express antimicrobial resistance genes.

The goal of this study was to compare the integron structure and gene-cassette content, plasmid profiles, and pulsed-field electrophoresis (PFGE) patterns of recent *S.* Typhimurium DT104 (R-type ACSSuT) isolates (1996) with the earliest identified isolate of MR-DT104, collected in 1985, as well as isolates from 1990. The resulting data would suggest whether the emergence of MR-DT104 in the mid-1990s in humans and animals resulted from the dissemination of a strain already present in the United States or from the introduction of a new strain.

Seven *S.* Typhimurium MR-DT104 strains isolated from ill persons in 1985 (one isolate), 1990 (three isolates), and 1996 (three isolates) were characterized by polymerase chain reaction (PCR), PFGE, and plasmid profile analysis. MR-DT104 isolates used in this study (Table) were sent to CDC by local and state health departments and public health laboratories in 1985, 1990, and 1996 as part of national surveys designed to assess the emergence of antimicrobial resistance in *Salmonella* isolates from humans in United States (*16*,*17*). These isolates were serotyped by using a modified version of the method described by Ewing (*18*). Sensitivity testing of chosen isolates (MICs) was conducted by using a panel of 17 antimicrobial agents (amikacin, amoxicillin-clavulanic acid, ampicillin, apramycin, ceftiofur, ceftriaxone, cephalothin, chloramphenicol, ciprofloxacin, gentamicin, kanamycin, nalidixic acid, streptomycin, sulfamethoxazole, tetracyline, ticarcillin, and trimethoprim-sulfamethoxazole) on the Sensititre system (TREK Diagnostic Systems, Westlake, OH). Phage typing was done by using the methods of the Laboratory of Enteric Pathogens, Central Public Health Laboratory, Public Health Laboratory Service, United Kingdom (*19*).

**Table:** Molecular characterization of multidrug-resistant *Salmonella* Typhimurium DT104 isolates, 1985–1996

Isolate number	Year	R-type	PFGE pattern	Plasmid content	Amplicons	Gene cassette(s)	*flo*-like gene^a^
B1579	1985	ACSSuT	1	60 mDa	1 kb; 1.2 kb	*ant(3")-Ia; psE1*	+
C3591	1990	ACSSuT	1	60 mDa	1 kb; 1.2 kb	*ant(3")-Ia; psE1*	+
C4501	1990	ACSSuT	1	60 mDa ~5 mDa	1 kb; 1.2 kb	*ant(3")-Ia; psE1*	+
C5234	1990	ACSSuT	1b	60 mDa	1 kb; 1.2 kb	*ant(3")-Ia; psE1*	+
G10217	1996	ACSSuT	1	60 mDa 3 mDa	1 kb; 1.2 kb	*ant(3")-Ia*; *psE1*	+
G10518	1996	ACSSuT	1	60 mDa	1 kb; 1.2 kb	*ant(3")-Ia; psE1*	+
G10551	1996	ACSSuT	1	60 mDa	1 kb; 1.2 kb	*ant(3")-Ia; psE1*	+

^a^All integrons are located on the chromosome**.** A = ampicillin; C = chloramphenicol; S = streptomycin; Su = sulfamethoxazole; T = tetracycline; PFGE = pulsed-field gel electrophoresis.

PFGE analysis was carried out as described by Barrett et al. (*20*), with the following modifications. The DNA in agarose plugs was restricted with 40 U/plug-slice of *Xba*I restriction enzyme (Roche, Indianapolis, IN), following the manufacturer’s recommendations. Restriction fragments were separated by PFGE through 1% SeaKem Gold agarose gels (BioWhittaker, Rockland, ME) in 0.5X Tris-borate-EDTA (45 mM Tris borate, 1 mM EDTA, pH 8.3 [TBE]) buffer at 14°C in a CHEF Mapper (Bio-Rad Laboratories, Hercules, CA). Electrophoresis conditions were as follows: initial switch time of 2.16 seconds, final switch time of 63.8 seconds, 6 V/cm, at an angle of 120° for 19 hours. The PFGE profiles (*Xba*I) of these isolates were indistinguishable from each other, with the exception of isolate C5234, which showed an extra band (indicated with an arrow in the Figure) compared with the other isolates. This level of clonality is remarkable considering that some of the isolates were obtained 11 years apart. The predominant PFGE pattern (Figure) was also seen in 21 of 22 additional MR-DT104 R-type ACSSuT isolates from humans in 1996 tested in our laboratory (E. Ribot, unpub. data). The remaining isolate had a PFGE pattern indistinguishable from the pattern of isolate C5234.

**Figure:**
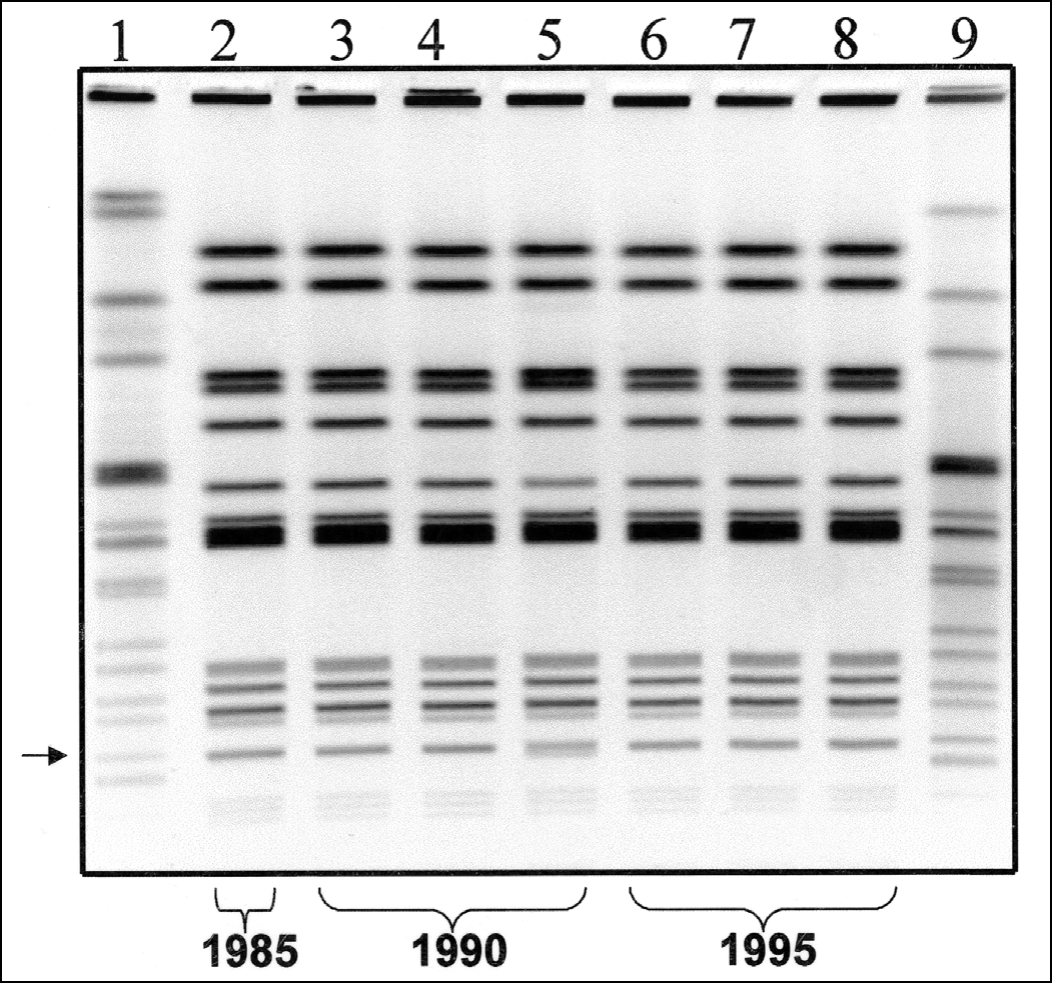
Gel showing the *Xba*I pulsed-field gel electrophoresis (PFGE) pattern of each isolate. Lane 1 and 9 show the pattern of the *Salmonella* strain used as a size standard. Lane 2 shows the pattern of MR-DT104 strain isolated in 1985. Lanes 3-5 show the patterns of MR-DT104 isolates obtained in 1990. Lanes 6-8 show the PFGE patterns of MR-DT104 isolates obtained in 1996.

Plasmid DNA was isolated by using the QIAfilter plasmid midi kit (Qiagen, Chatsworth, CA), following the manufacturer’s instructions. Plasmid profile analysis was done by loading 15- to 20-µL aliquots into the wells of 1% SeaKem Gold agarose gels and performing PFGE. The pulsing conditions were as follows: electrophoresis for 16 hours on a CHEF Mapper or GenePath System (Bio-Rad), with an initial switch time 6.75 seconds and final switch time 21.7 seconds at 6V/cm. The running buffer consisted of 0.5X TBE. The buffer temperature was kept at 14°C during the PFGE run. The plasmid patterns were visualized by ethidium bromide staining. Plasmid profile analysis showed that all *S.* Typhimurium MR-DT104 isolates contained the 60-mDa plasmid commonly seen in this strain (data not shown). Additional, smaller plasmids were observed in some of the isolates (Table). The nature or function of these smaller plasmids is not known.

Multidrug resistance is associated with the presence of two integrons found in the chromosomal DNA of *S.* Typhimurium MR-DT104 R-type ACSSuT isolates (*2*,*8*,*9*). To investigate the possibility that these integrons were also present in the *S.* Typhimurium DT104 R-type ACSSuT isolates from 1985 and 1990, the first MR-DT104 isolates found in our collection, we used PCR to amplify gene-cassettes located between the two conserved regions (5´-CS and 3´-CS) of class-1 integrons. To accomplish this, genomic DNA was isolated by using the Puregene DNA isolation kit (Gentra Systems, Minneapolis, MN), following the manufacturer’s recommendations. PCR amplification was performed on both chromosomal DNA and purified plasmid preparations with synthetic oligonucleotides 5´-CS (5´-GGCATCCAAGCAGCAAG-3´) and 3´-CS (5´-AAGCAGACTTGACCTGA-3´) (*12*). PCR was also used to amplify the region located between the 5´CS and the sulI (SulI: 5´-TGAAGGTTCGACAGCAC-3´) (*12*). The cycling conditions consisted of an initial step at 94°C for 10 minutes, followed by 35 cycles at 94°C for 20 seconds, 56°C for 20 seconds, and 72°C for 45 seconds with a final extension step of 10 minutes at 72°C. PCR reactions were performed by using Taq Gold polymerase (PE Biosystems, Foster City, CA) or Taq DNA polymerase (Roche), under the reactions conditions recommended by the manufacturers. A PCR System 2400 (PE Biosystems) was used for all PCR amplifications. The resulting PCR amplicons were loaded onto a 1% SeaKem Gold agarose gel and subjected to electrophoresis at 80 volts for 1 hour and visualized by ethidium bromide staining.

The PCR amplification with the 5´-CS:3´-CS primer set yielded two products, a 1-kb and a 1.2-kb fragment (data not shown). These two fragments represent the gene cassettes contained within two different integrons. The PCR products were purified by using the QIAquick PCR purification kit (Qiagen). No amplicons were obtained from any of the plamid preparations, confirming the chromosomal nature of the antimicrobial resistance. The purified products were sequenced by the dye terminator reaction method described in ABI Prism 377 DNA sequencer (PE Biosystems) instruction manual. DNA sequence analysis was carried out by using the FASTA algorithm of the GCG Wisconsin package.

Computer analysis showed that the 1-kb fragment contained a gene homologous to the ant(3′′)-1a gene, a gene that confers resistance to streptomycin and spectinomycin. The 1.2-kb PCR amplicon contained the psE1 gene, which encodes a beta-lactamase that confers resistance to ampicillin. PCR ampification with oligonucleotides specific for the 5´-CS and *sulI* gene yielded a product of the expected size (approximately 1.1 kb larger than the product obtained with the 5´-CS:3´-CS primer set), indicating the presence of *sulI*, which confers resistance to sulfonamides.

We were also interested in determining whether resistance to chloramphenicol was due to the presence of the *flo*-like gene described by Bolton et al. in 1999 (21). Detection of *flo* was carried out by using the oligonucleotide primers, *flo*-1 (5´-AATCACGGGCCACGCTGTATC-3´) and *flo*-2 (5´-CGCCGTCATTCTTCACCTTC-3´) (21). PCR and sequencing data confirmed that all the isolates carried the florfenicol resistance (*flo*-like) gene (data not shown). These findings are consistent with those of earlier reports (*7*–*9*,*21*) and further demonstrate the high degree of clonality among isolates of *S.* Typhimurium MR-DT104 dating back to 1985.

Our data suggest that the strain of MR-DT104 that became prevalent during the mid-1990s had not caused frequent human illness in the United States in 1985 and 1990. Local health officials interviewed the four patients from whom these MR-DT104 isolates were collected in 1985 and 1990; none of them reported traveling outside the United States 30 days before onset of illness. None reported other underlying illnesses, and none reported taking any antibiotics before specimens were collected. These data suggest that domestic transmission of MR-DT104 to humans, perhaps through contaminated food, occurred in 1985 and 1990. Human infection with MR-DT104 did not become prevalent, however, until the mid-1990s. The factors that led to the widespread dissemination of MR-DT104 in humans in the United States in the mid-1990s are unknown.

However, the limited animal data available indicate that MR-DT104 became disseminated in food animals at approximately the same time (*6*). Factors that contributed to the dissemination of MR-DT104 in animals are poorly understood. Since food animals are the reservoir for most domestically acquired human *Salmonella* infections and transmission from animals to humans occurs through the food supply (*5*), the rapid dissemination of MR-DT104 among humans in the mid-1990s was likely the consequence of dissemination of MR-DT104 in food animals during the same period. If this hypothesis is correct, it would parallel the experience of the United Kingdom, where MR-DT104 was identified as early as 1984 but did not become epidemic in humans until it was established in cattle in the late 1980s (*2*).

Molecular evidence supports the suggestion that some of the antimicrobial resistance determinants found in *S.* Typhimurium DT104 R-type ACSSuT may have emerged, perhaps in Asia, in the early 1980s in other bacteria and were transferred horizontally to DT104 (*5*). For example, chloramphenicol resistance in MR-DT104 is encoded by a *flo*-like gene that confers resistance to both chloramphenicol and florfenicol (*21*,*22*). *flo* was first identified in *Pasteurella piscicida*, the causative agent of pseudotuberculosis, a common disease of marine fish in Asia (*23*). Florfenicol was evaluated as a therapeutic agent in fish in Asia in the early 1980s (*22*–*26*). Kim et al. (*27*) reported the emergence of florfenicol-resistant strains of *P. piscicida* due to the acquisition of transferable resistance plasmid containing the *flo* gene, in addition to other antimicrobial resistance markers (ampicillin, kanamycin, sulfonamide, and tetracycline). Furthermore, nucleotide sequence analysis of the DNA region containing the florfenicol resistance gene and the two tetracycline genetic determinants showed a 94% similarity to a sequence found in a plasmid from *P. piscicida* (*22*). Additional evidence supporting the idea of horizontal transfer of multiple antimicrobial resistance determinants comes from a recent study conducted on multidrug resistant *S.* Agona isolates containing a DT104-like antimicrobial resistance gene cluster in their genome (*28*).

In summary, MR-DT104 isolates with indistinguishable PFGE patterns and carrying the same or highly similar integrons as more recent isolates were present in the United States as early as 1985. These data and the fact that food-producing animals are the reservoirs for most human *Salmonella* infections in the United States suggest that the emergence of MR-DT104 during the mid-1990s probably resulted from the dissemination of MR-DT104 in food-producing animal reservoirs. Laboratory and epidemiologic evidence is insufficient to determine whether this dissemination resulted from spread of a strain already present in the United States or the reintroduction of the strain through importation of contaminated livestock or other means.
